# Untangling the oxidative cost of reproduction: An analysis in wild banded mongooses

**DOI:** 10.1002/ece3.8644

**Published:** 2022-03-08

**Authors:** Magali Meniri, Elsa Evans, Faye J. Thompson, Harry H. Marshall, Hazel J. Nichols, Gina Lewis, Lauren Holt, Emma Davey, Christopher Mitchell, Rufus A. Johnstone, Michael A. Cant, Jonathan D. Blount

**Affiliations:** ^1^ College of Life & Environmental Sciences Centre for Ecology & Conservation University of Exeter Penryn UK; ^2^ 4920 Whitelands College Centre for Research in Ecology, Evolution & Behaviour University of Roehampton London UK; ^3^ 7759 Department of Biosciences Swansea University Swansea UK; ^4^ 2152 Department of Zoology University of Cambridge Cambridge UK

**Keywords:** constraint, cost, *Mungos mungo*, oxidative stress, reproduction, shielding

## Abstract

The cost of reproduction plays a central role in evolutionary theory, but the identity of the underlying mechanisms remains a puzzle. Oxidative stress has been hypothesized to be a proximate mechanism that may explain the cost of reproduction. We examine three pathways by which oxidative stress could shape reproduction. The “oxidative cost” hypothesis proposes that reproductive effort generates oxidative stress, while the “oxidative constraint” and “oxidative shielding” hypotheses suggest that mothers mitigate such costs through reducing reproductive effort or by pre‐emptively decreasing damage levels, respectively. We tested these three mechanisms using data from a long‐term food provisioning experiment on wild female banded mongooses (*Mungos mungo*). Our results show that maternal supplementation did not influence oxidative stress levels, or the production and survival of offspring. However, we found that two of the oxidative mechanisms co‐occur during reproduction. There was evidence of an oxidative challenge associated with reproduction that mothers attempted to mitigate by reducing damage levels during breeding. This mitigation is likely to be of crucial importance, as long‐term offspring survival was negatively impacted by maternal oxidative stress. This study demonstrates the value of longitudinal studies of wild animals in order to highlight the interconnected oxidative mechanisms that shape the cost of reproduction.

## Graphical abstract

The cost of reproduction plays a central role in evolutionary theory, but the identity of the underlying mechanisms remains a puzzle. Oxidative stress has been hypothesized to be a proximate mechanism that may explain the cost of reproduction. We examine three pathways by which oxidative stress could shape reproduction using data from a long‐term food provisioning experiment on wild female banded mongooses (*Mungos mungo*).

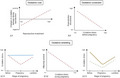

## 1 INTRODUCTION

What mechanisms may limit an individual's ability to reproduce? This central question in evolutionary biology has intrigued scientists for decades (Harshman & Zera, 2007; Linden & Møller, 1989; Reznick, 1985). Oxidative stress has been proposed as an important mechanism that might underlie trade‐offs in current reproduction, as well as with future reproduction and/or survival (Dowling & Simmons, 2009; Metcalfe & Alonso‐Alvarez, 2010; Monaghan et al., 2009). When reactive oxygen species (ROS), produced as a by‐product of metabolism, overwhelm the body's antioxidant machinery that functions to neutralize ROS, the body enters a physiological state of oxidative stress. ROS can cause serious damage to biomolecules such as proteins, lipids, and DNA, and may ultimately impair cell homeostasis and function (reviewed by Halliwell & Gutteridge, 2007; Speakman et al., 2015). Oxidative stress can thus have highly detrimental consequences on virtually every life history trait, from growth to aging (Metcalfe & Alonso‐Alvarez, 2010; Monaghan et al., 2009; Speakman et al., 2015).

Researchers have reported a variety of associations between reproduction and oxidative stress. First, multiple studies have found a positive correlation between reproductive effort (offspring number or size) and oxidative stress (zebra finches *(Taeniopygia guttata)*: Bertrand et al., 2006; Wiersma et al., 2004; house mice *(Mus musculus)*: Garratt et al., 2011; Plumel et al., 2014; Eastern chipmunks (*Tamias striatus*): Bergeron et al., 2011; Brandt's voles (*Lasiopodomys brandtii*): Xu et al., 2014; and common lizards (*Zootoca vivipara*): Dupoué et al., 2020). According to the “*oxidative cost”* hypothesis, such an association is expected because reproductive investment may result in increased metabolic rate and, consequently, elevated ROS production. Evidence suggests that the relationship between metabolic rate and ROS production is not linear, and indeed in some circumstances energy turnover and ROS production can be negatively correlated, notably due to mitochondrial uncoupling (Salin et al., 2015; Speakman & Garratt, 2014). Indeed, oxidative phosphorylation, a major source of ATP, uses a series of redox reactions to create an electrochemical proton gradient between the inner mitochondrial membrane and the matrix in order to synthesize ATP. During this process, electrons used to build the proton gradient can leak and lead to the formation of ROS. However, uncoupling proteins can partly dissipate this gradient by leaking protons, thus lowering the efficiency of oxidative phosphorylation, and in turn decreasing ROS production, in a process known as mitochondrial uncoupling (Cadenas, 2018). Nevertheless, periods of high energy requirements and thus high metabolic activity such as growth and reproduction can represent an oxidative challenge, as numerous studies have shown (growth: Janssens & Stoks, 2020; Smith et al., 2016, reproduction: Bertrand et al., 2006; Bergeron et al., 2011; Dupoué et al., 2020; Garratt et al., 2011; Plumel et al., 2014; Wiersma et al., 2004). Indeed, mitochondrial uncoupling reduces the efficiency of ATP production, and as such seems unlikely to occur during reproduction, when energy demands are particularly high. In mammals, for example, lactation is the most energetically costly period of reproduction (Speakman, 2008).

Second, evidence from several taxa suggests that individuals with higher levels of oxidative stress prior to reproduction subsequently have lower reproductive output (house mice: Stier et al., 2012; canaries (*Serinus canaria)*: Costantini et al., 2016; and brown boobys *(Sula leucogaster*): Montoya et al., 2016). Thus, oxidative stress might constrain an individual's capacity to invest in reproduction, potentially to avoid excessively high oxidative costs of reproduction that could damage fitness. This has been coined the “*oxidative constraint*” hypothesis and focusses on intra‐generational costs of reproduction.

Finally, when considering reproductive state, some studies have found higher levels of oxidative stress in breeders compared to non‐breeders (Asp vipers (*Vipera aspis*): Stier et al., 2017), while most studies have surprisingly found the opposite pattern (house mice: Garratt et al., 2011; bank voles (*Myodes glareolus*): Ołdakowski et al., 2012; Ołdakowski et al., 2015; Damaraland mole‐rats (*Fukomys damarensis*): Schmidt et al., 2014; canaries: Costantini et al., 2014; banded mongooses (*Mungos mungo)*: Vitikainen et al., 2016; and Columbian ground squirrels (*Urocitellus columbianus*): Viblanc et al., 2018), or no significant association between reproductive state and oxidative stress (zebra finches: Bertrand et al., 2006). Such inconsistencies led some researchers to highlight potential shortcomings in the design of previous studies (Metcalfe & Monaghan, 2013; Speakman & Garratt, 2014), while others have questioned the existence of a proximate link between oxidative stress and reproduction (Ołdakowski et al., 2015; Speakman & Garratt, 2014). However, results of a recent meta‐analysis have shown that, overall, breeders exhibit lower levels of oxidative stress compared to non‐breeders (Blount et al., 2016), which highlights that this pattern is more widespread than previously thought. It has been suggested that individuals might pre‐emptively decrease oxidative stress levels before they reproduce, in order to shield themselves, and their physiologically dependent offspring from negative intergenerational consequences of oxidative stress during reproduction (*“oxidative shielding”* hypothesis) (Blount et al., 2016). Interestingly, a previous study conducted on banded mongooses has found evidence that supports the oxidative shielding hypothesis, with breeders displaying lower levels of oxidative damage to lipids compared to non‐breeders. Moreover, that same study, along with a few others, have found that maternal oxidative stress during breeding can negatively impact offspring production and development (Bize et al., 2008; Dupoué et al., 2020; Essa et al., 2015; Møller et al., 2008; Vitikainen et al., 2016). Although mechanisms of shielding are not yet well understood, presumably mothers incur some costs through damage reduction (e.g., by upregulation of antioxidant defenses); otherwise, it would be expected that oxidative damage should be maintained at low levels all of the time.

Thus, oxidative stress may shape reproduction in various ways: via oxidative costs, oxidative constraints, and oxidative shielding. However, these three mechanisms have rarely been explored in parallel (but see Viblanc et al., 2018), and it remains unclear whether oxidative constraint and shielding represent complementary, or alternative paths to optimize lifetime reproductive success. It seems possible that these mechanisms might co‐occur. However, the only study to have tested these three hypotheses to date found support for the shielding hypothesis (Viblanc et al., 2018). Using a wild population of Columbian ground squirrels, these authors found that breeding females displayed higher levels of antioxidants and lower oxidative damage during lactation compared to levels at birth, and compared to non‐breeding females, but little evidence was found for an oxidative cost or oxidative constraint on reproduction.

An interesting question is why, and in what circumstances, a mother adopts one strategy instead of another, between constraining her investment into reproduction and exhibiting shielding to optimize breeding success while minimizing oxidative stress levels. The nutritional condition of mothers could play an important role. Antioxidants are diverse, and include both diet‐derived compounds such as vitamin E and endogenously produced molecules such as the enzyme superoxide dismutase (SOD) and the peptide glutathione (GSH) (reviewed by Halliwell and Gutteridge (2007)). Improved nutrition may therefore allow mothers to allocate more resources toward antioxidant defenses. This could occur both by acquiring exogenous antioxidants and by providing more resources for individuals to synthesize endogenous antioxidants. Specifically, glutathione, although being endogenously produced, requires specific nutrient precursors for its synthesis such as the amino acid cysteine and methionine (Lu, 2013). Chickens *(Gallus gallus domesticus)* supplemented with methionine exhibited higher levels of glutathione (Németh et al., 2004). More generally, in North American red squirrels (*Tamiasciurus hudsonicus*) supplemental feeding led to higher antioxidant defenses and lower oxidative damage levels (Fletcher et al., 2013), while in great tits (*Parus major*) experimentally provisioned individuals exhibited lower oxidative damage levels compared to control individuals (Giordano et al., 2015). Therefore, improved nutrition may potentially increase offspring production while also ensuring that oxidative stress does not exceed a threshold that would damage fitness. In order to understand how oxidative stress shapes reproduction, it is necessary to follow individuals before and during breeding, and to examine associations among maternal oxidative stress markers, investment in offspring production for each litter, and the development and survival of offspring. In addition, it is important to investigate how changes in maternal nutrition may alter oxidative state and patterns of reproductive investment.

In order to gain a better understanding of the interplay among oxidative stress, reproduction, and maternal nutrition, we conducted a long‐term (up to 2.8 years) food provisioning experiment using wild, female banded mongooses. Banded mongooses live in mixed‐sex social groups comprising 5–25 adults (Cant et al., 2013, 2016). Each social group breeds on average four times a year (Cant, 2000). Breeding females in the same social group give birth synchronously, and all adults communally raise the offspring (Cant, 2000). This breeding system therefore allows for powerful split‐plot experiment designs, where comparisons can be made between provisioned and control individuals, while breeding synchronously and in exactly the same environment.

We predicted that (1) compared to non‐provisioned controls, experimental provisioning would allow females to:

(1.1) Allocate more resources toward antioxidant defense, and thus exhibit lower oxidative damage levels;
(1.2) Increase offspring production per litter by allowing higher pre‐natal investment and/or offspring survival.

**FIGURE 1:**
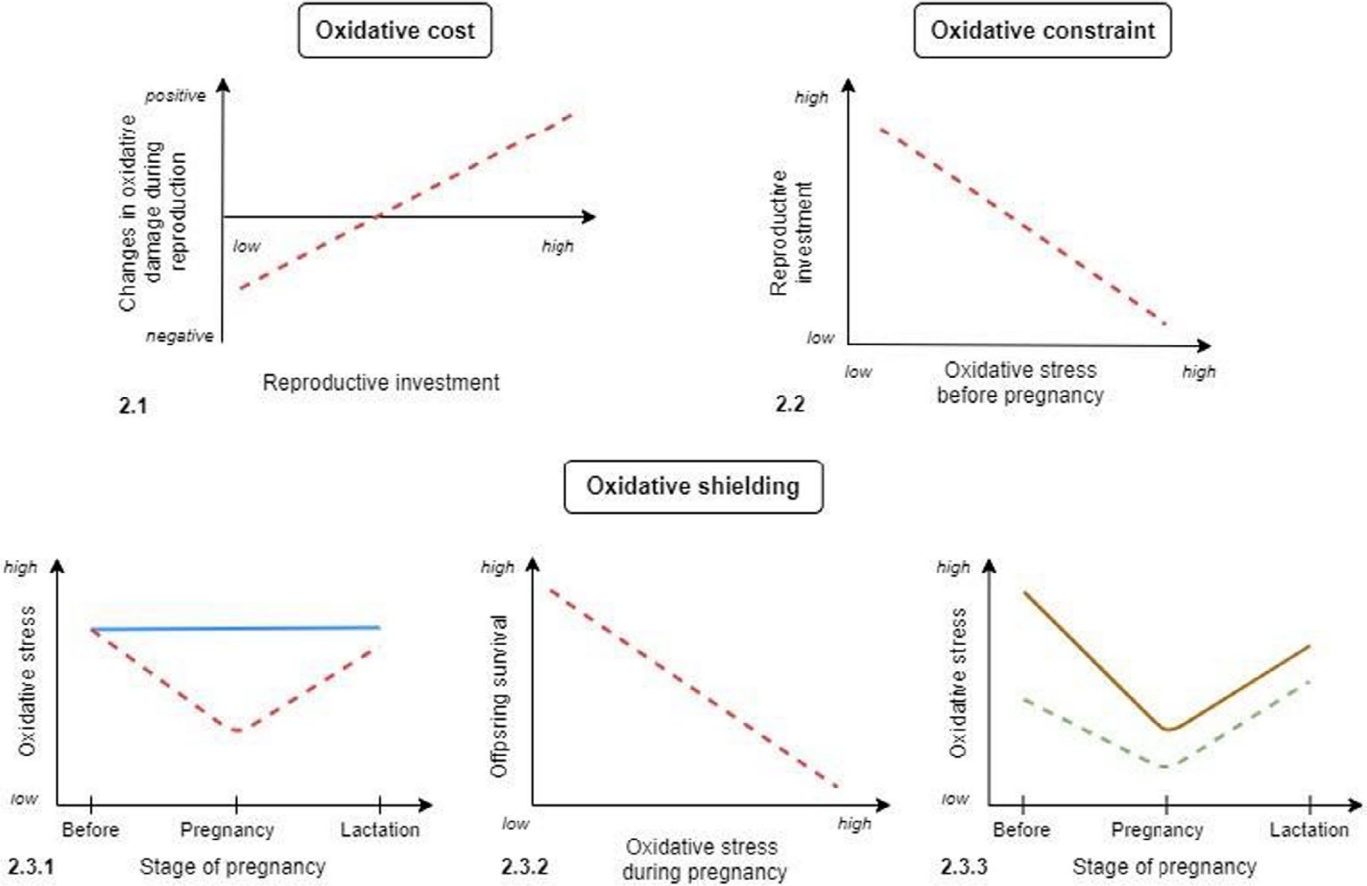
Predictions associated with each hypothesis. 2.1: Oxidative cost, 2.2: Oxidative constraint, and 2.3: Oxidative shielding. Red dashed lines represent breeders, blue solid line represents non‐breeders. For 2.3.3, the orange solid line represents individuals that exhibit high levels of oxidative stress before breeding, while the green dashed line represents individuals that exhibit low levels of oxidative stress before breeding

We also (2) aimed to explore how oxidative stress shapes reproduction. We predicted that (Figure 1):

(2.1) *Oxidative cost*: There would be a positive association between levels of reproductive effort (i.e., offspring number and/or size) and subsequent increase in levels of oxidative damage;
(2.2) *Oxidative constraint*: Females with higher levels of oxidative damage before reproduction would subsequently invest less in offspring production;
(2.3) *Oxidative shielding*:
  (2.3.1) Breeding individuals would exhibit a within‐individual decrease in oxidative damage during breeding, leading to lower levels in breeders compared to non‐breeders during pregnancy;
  (2.3.2) Maternal levels of oxidative stress during pregnancy would be negatively correlated with reproductive investment and/or offspring survival; and
  (2.3.3) Individuals exhibiting higher levels of oxidative damage before reproduction would exhibit the steepest decrease in oxidative damage during reproduction.

## 2 MATERIALS AND METHODS

### 2.1 Study population

We collected data from a wild population of banded mongooses on the Mweya Peninsula, Queen Elizabeth National Park, Uganda (0°12′S, 29°54′E). Detailed life history data on this population have been collected continuously since 1995 (Cant et al., 2013, 2016). Typically, our study population consists of 10–12 social groups that are visited every 1–3 days to record group composition, life history, and behavioral data. Banded mongooses always disperse in groups, making it possible to unequivocally distinguish death from dispersal (Cant et al., 2001). Most individuals are trained to step onto a portable electronic balance in return for a small milk reward, and are weighed weekly in the field before morning foraging. Groups containing pregnant females are visited daily to obtain accurate birth dates. Gestation lasts on average 60 days (Cant, 2000). Individuals in the population are identified using unique shave markings on their back, and PIT tags (TAG‐P‐122IJ, Wyre Micro Design Ltd., UK) inserted under the skin on the scruff of the neck. Pups are trapped within 2 weeks of emergence from the den (between 30 and 50 days of age) and anesthetized using isoflurane. They are then weighed, measured, and marked using commercially available blonde hair dye (L'Oreal, UK). A ~2 mm skin sample is collected from the tail tip for genetic assignment of maternity (Sanderson et al., 2015). Individuals within the population are trapped every 3–6 months, using box traps (67 × 23 × 23 cm; Tomahawk Live Trap Co., Tomahawk, WI, USA), and anesthetized using isoflurane prior to measurements of morphometrics, ultrasound scans, and collection of blood samples.

### 2.2 Experimental provisioning of females

Experimental provisioning was conducted in six groups between May 2017 and March 2020. Provisioned females were fed one egg, gently cooked as an omelet, three times a week. Each provisioned female was associated with an age‐matched within‐group control female that remained non‐provisioned. If an experimental female died, another female from the group was selected as a replacement. A total of 18 females were provisioned, and 15 females acted as controls, for a mean duration of 561 days (min = 165 days; max = 1053 days). A total of 71 litters were born during the experimental period, with an average of 6.7 litters per female (min = 2; max = 15).

### 2.3 Ultrasound scanning

We carried out ultrasound scans of fetuses carried by pregnant females to measure pre‐natal offspring production. The ultrasound scans were taken around day 25 of pregnancy (mean ± SE = 25.29 ± 0.67 days). A Sonoscape S6BW ultrasound scanner with a L742 linear probe (Vet Image solutions, UK) was used to obtain cross‐sectional images of each fetus along their transverse plane in their gestational sac at their widest point. Scans were not used if the image was unclear, if the fetus was cut off the edge of the image, or if the gestational sacs were not elliptical in shape. Perpendicular measurements of the gestational sac were taken using ImageJ (Schneider et al., 2012), where measurement “B” was taken along the longest axis of the gestational sac at 90° from measurement “A” (see example in Figure S1). The cross‐sectional area was then calculated following the methods of Inzani et al. (2016) using the formula: cross‐sectional area = (*A*/2) × (*B*/2) × *π*.

### 2.4 Pre‐natal investment

An index of pre‐natal investment was computed by calculating the mean fetus size measured *in utero* for each female for a given pregnancy using ultrasound scans, multiplied by the number of fetuses carried by each female. To account for differences in the exact day the scan was taken compared to date of birth, fetus size was divided by the age at measure relative to date of birth. We used the mean fetus size for a given pregnancy for each female, as it was sometimes not possible to measure accurately the size of each fetus.

### 2.5 Collection of blood samples

Blood (volume 100–500 μl) was collected from the jugular vein using a 25G needle and syringe, and transferred to a 3‐ml EDTA BD Vacutainer^®^. Whole blood was centrifuged at 2000 x g for 4 min at 4°C (Spectrafuge mini centrifuge, Sigma Aldrich, UK) to separate the plasma, which was frozen for analyses of malondialdehyde (MDA) and protein carbonyls (PC). Samples of red blood cells (RBC) were frozen for analysis of glutathione (GSH) and superoxide dismutase (SOD). All samples were snap frozen in liquid nitrogen within 10 min of collection, and subsequently transported to our UK laboratory in a cryogenic shipper (Taylor‐Wharton CX100, Jencons, UK) and stored at −80°C until analysis.

Sampling occurred *before pregnancy*: between 80 days and 60 days before the litter's birth date [mean ± SE = −66.5 ± 0.6 days]; *during pregnancy*: between 49 days and 0 days before the litter's birth date [mean ± SE = −24.8 ± 0.78 days]; or *during lactation*: between the litter's birth date and 30 days afterwards [mean ± SE = 12.35 ± 0.54 days], as weaning occurs at 40 days.

### 2.6 Quantification of oxidative stress markers

Four oxidative stress markers were selected based on their biological importance. MDA and PC are two major markers of oxidative damage, to lipids and proteins, respectively. In addition, SOD and GSH are two major endogenous antioxidants that are often found to be of crucial importance in maintaining oxidative balance. These antioxidants act at two different stages in the neutralization of ROS, with SOD first catalyzing the dismutation of the superoxide anion into oxygen and hydrogen peroxide, whereas glutathione decomposes hydrogen peroxide into water by oxidizing the reduced form of glutathione (GSH) into the oxidized form of glutathione (GSSG). Sample size was sometimes limiting: for plasma, quantification of MDA was favored over PC; for RBC, GSH, and SOD, quantification requires low sample volumes, so both were quantified for each sample.

Lab analyses were performed blindly with respect to sample identity, and all steps were conducted on ice. All chemicals were HPLC grade, and chemical solutions were prepared using Milli‐Q water (Milli‐Q Synthesis; Millipore, Watford, UK). Assays were conducted within 1 year of collection (time since collection (mean ± SE): MDA: 268 ± 89 days; PC: 295 ± 94 days; SOD: 286 ± 58 days; and GSH: 295 ± 60 days).

Plasma malondialdehyde (MDA), a marker of lipid peroxidation, was determined using an HPLC with a fluorescence detector (Agilent 1000; Agilent Technologies, USA). We followed the method in Nussey et al. (2009) with some modifications. Details can be found in the Supplementary Material. MDA level in sample is expressed in μM; the coefficient of variation for 88 duplicate samples was 9.5%.

Plasma protein carbonyls (PC), a marker of protein oxidative damage, were measured using a colorimetric assay following a protocol adapted from the Carbonyl Assay Kit (Cayman Chemical Company, USA). Details can be found in the Supplementary Material. Carbonyl content in samples is expressed in nmol/mg protein; the coefficient of variation for 65 duplicate samples was 12.5%.

We assessed superoxide dismutase (SOD) activity (U/ml), an endogenous enzymatic antioxidant, in RBC samples using the Cayman Chemical Superoxide Dismutase Assay Kit (Cayman Chemical Company, USA). Details can be found in the Supplementary Material. The coefficient of variation computed for 40 duplicate samples was 10.8%.

We assessed reduced glutathione (GSH) level, an endogenous antioxidant, in RBC samples using the Cayman Chemical Glutathione Assay Kit (Cayman Chemical Company, USA). Details can be found in the Supplementary Material. Reduced glutathione level is expressed in μM; the coefficient of variation computed for 38 duplicate samples was 11.9%.

### 2.7 Statistical analyses

Data were analyzed using R, version 3.6.1 (R Core Team, 2017). The “lme4” package was used for linear mixed‐effects models (Bates et al., 2019), while the “lmerTest” package was used to obtain *p*‐values (Kuznetsova et al., 2017). Non‐significant interactions were not removed, as Type II sums of squares were used for the ANOVA, which does not assume the presence of an interaction to estimate main effects (Langsrud, 2003). The “stats” package was used for generalized linear models (R Core Team, 2017), while the “coxme” package was used to run survival mixed‐effects Cox models (Therneau, 2020). The “emmeans” package was used to perform post hoc tests, with false discovery rate correction for test multiplicity (Lenth et al., 2020). For all analyses, we checked model assumptions, i.e., normality, linearity, homoscedasticity, and proportional hazards for survival analysis. The significance level was set at 0.05. When required, litter identity was included as a random factor to control for any litter effects. Maternal identity was included to avoid pseudo‐replication, as mothers may have had several pups within the same litter, or may have participated in several litters.

An interplay is likely to occur among maternal nutrition, oxidative stress, and reproduction. However, testing for an interaction between maternal provisioning treatment and reproductive investment/offspring survival or oxidative stress markers would be statistically dubious. Indeed, we predicted that the provisioning treatment would impact both oxidative stress markers and maternal investment/offspring survival. Therefore, including both the provisioning treatment and oxidative stress markers or the provisioning treatment and reproductive investment/offspring survival as explanatory variables in the same model would result in high risk of multicollinearity, as checked using the variance inflation factor (VIF), which represents a major issue for the interpretation of linear models (Kraha et al., 2012). Therefore, we first examined the effect of the treatment on maternal oxidative stress markers and on reproductive investment/offspring survival. Then, we examined oxidative stress data to understand the impact on maternal investment/offspring survival, and thus evaluate whether oxidative stress shaped reproduction.

#### 2.7.1 Consequences of maternal provisioning experiment

We tested the effect of maternal provisioning treatment on oxidative stress marker dynamics during the breeding event in pregnant females. To do so, we ran linear mixed‐effect models with oxidative stress markers (PC, MDA, SOD, or GSH) as a response variable, with timing of measurement (*before pregnancy*, *during pregnancy*, or *during lactation*), maternal provisioning treatment, and their interaction as explanatory variables. Litter identity and maternal identity were included as random effects. Day of sampling (relative to date of birth) was initially included as a covariate. However, it was never significant, and as such was removed from the final models to keep them as simple as possible.

We did not use data reduction methods such as PCA with oxidative markers for two reasons. First, we did not have oxidative stress measures for all markers for each individual because of blood sample volume limitations. Second, the markers of oxidative stress were only very weakly correlated. Indeed, MDA showed a Pearson's correlation coefficient of *r* = −.004 (*p*‐value = .95) with PC, *r* = −.08 (*p*‐value = .16) with SOD, and *r* = −.08 (*p*‐value = .15) with GSH. SOD showed a correlation coefficient of *r* = −.06 (*p*‐value = .31) with PC and *r* = −.10 (*p*‐value = .08) with GSH, while PC and GSH had a correlation of *r* = .06 (*p*‐value = .28). Given such weak correlations, PCA did not provide easily interpretable principal components. Therefore, we decided to use individual markers in our models.

To examine the effect of the maternal provisioning treatment on maternal investment and offspring survival, we ran linear mixed‐effect models with pre‐natal investment, offspring body mass at emergence from the den, or number of offspring emerging from the den as a response variable, and maternal provisioning treatment as an explanatory variable. For the model with offspring body mass at emergence as a response variable, to account for differences in the age at which body mass was measured, age at measurement was included as a covariate. For the model with number of offspring emerging from the den as a response variable, number of fetuses carried per female was included as a covariate. Litter identity and maternal identity were included as random effects.

The impact of the maternal provisioning treatment on survival to 12 months was determined using a Cox proportional hazard model, with survival to 12 months as a response variable, and the maternal provisioning treatment as the explanatory variable. Litter identity and maternal identity were included as random effects.

#### 2.7.2 How does oxidative stress shape reproduction?

To investigate how oxidative stress shapes reproduction, we used data from both provisioned and non‐provisioned females. To check whether reproduction represents an oxidative cost, we ran linear mixed models with within‐individual changes in levels of oxidative stress markers (PC, MDA, SOD, or GSH) during the breeding event as a response variable. Within‐individual changes were calculated as the difference between the marker levels during pregnancy and the levels before pregnancy. These differences were adjusted to account for potential regression toward the mean, a phenomenon where extreme values in a first measure are likely to be closer to the mean in a second measure. They were adjusted following Kelly and Price (2005) (see Supplementary Material for detailed formula). Pre‐natal investment and offspring body mass at emergence from the den were used as explanatory variables. To account for differences in the age at which body mass was measured, body mass was divided by the age at measurement. Litter identity and maternal identity were included as random effects.

To explore whether maternal investment in reproduction is constrained by maternal oxidative stress levels prior to reproduction, we ran two linear mixed models, with either pre‐natal investment or offspring body mass at emergence from the den as response variables, and all oxidative stress markers (PC, MDA, SOD, and GSH) measured before pregnancy as explanatory variables. For the model with offspring body mass at emergence as a response variable, to account for differences in the age at which body mass was measured, age at measurement was included as a covariate. Litter identity and maternal identity were included as random effects.

We investigated the oxidative shielding hypothesis, and specifically aimed to explore the effect of breeding status (breeders vs. non‐breeders) on oxidative stress markers dynamics during the breeding event. To do so, we ran linear mixed‐effect models with oxidative stress markers as a response variable (PC, MDA, SOD, or GSH) with time of measurement (*before pregnancy*, *during pregnancy*, or *during lactation*), breeding status, and their interaction as explanatory variables. Litter identity and maternal identity were included as random effects. For non‐breeders, time of measurement was assigned based on the date of the breeding event they belong to. Day of sampling (relative to date of birth) was initially included as a covariate. However, it was never significant, and as such was removed from the final models to keep them as simple as possible.

To check whether maternal oxidative stress levels were related to offspring fitness, we ran several linear mixed models. Pre‐natal investment, offspring body mass at emergence from the den, and number of offspring emerging from the den were used as a response variable, and all oxidative stress markers (PC, MDA, SOD, and GSH) measured during pregnancy as explanatory variables. For the model with offspring body mass at emergence as a response variable, age at measurement was included as a covariate to account for differences in the age at which body mass was measured. Litter identity and maternal identity were included as random effects.

The impact of maternal oxidative stress levels during pregnancy on survival to 12 months was investigated using a Cox proportional hazard model, with survival to 12 months as a response variable, and all oxidative stress markers (PC, MDA, SOD, and GSH) measured during pregnancy as explanatory variables. Litter identity and maternal identity were included as random effects. To explore whether individuals adjusted their oxidative stress levels during the breeding event based on their baseline levels, we ran linear mixed models with within‐individual changes in levels of oxidative stress markers (PC, MDA, SOD, or GSH) during the breeding event as a response variable, calculated as the difference between marker levels during pregnancy and levels before pregnancy. These differences were adjusted to account for regression toward the mean according to Kelly and Price (2005) (see Supplementary Material for more information on these adjustments). We used levels of oxidative stress markers before reproduction as explanatory variables, with body mass before pregnancy as a covariate, in an attempt to assess whether female's condition might influence that relationship. Litter identity and maternal identity were included as random effects.

## 3 RESULTS

### 3.1 Consequences of maternal provisioning experiment

**FIGURE 2:**
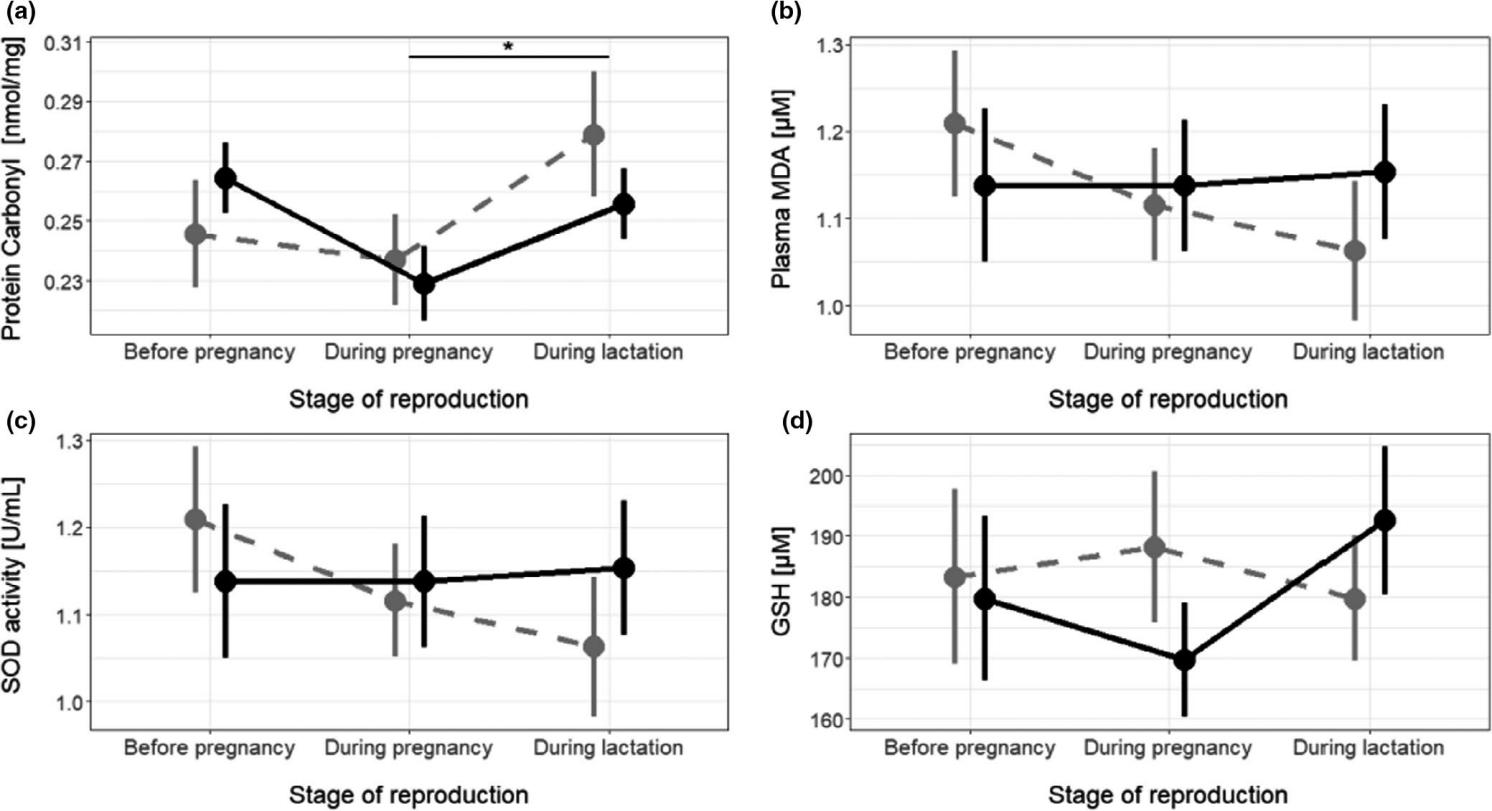
Dynamics of oxidative stress markers during the breeding event for breeders. (a) Protein Carbonyl level, (b) MDA level, (c) SOD activity, and (d) GSH level. Black dots and solid lines represent provisioned females, while grey dots and dashed lines represent non‐provisioned females. Symbols represent raw data means ± SE. Stars indicate statistical significance

In breeders, levels of protein carbonyls varied according to the stage of reproduction (Table 1), with post hoc tests showing lower levels during pregnancy compared to lactation (*T*‐ratio = −2.7, *p*‐value = .02). However, levels of protein carbonyls did not differ significantly between provisioned and non‐provisioned females, or according to the interaction between stage of reproduction and maternal provisioning treatment (Table 1, Figure 2). Levels of MDA, SOD, and GSH did not differ significantly in relation to the stage of reproduction, maternal provisioning treatment, or their interaction (Table 1, Figure 2).

**TABLE 1:** Test of prediction 1.1

	Protein carbonyl (nmol/mg protein)				MDA (μM)				SOD (U/ml)				GSH (μM)			
	*N*	Estimate ± SE	*F*‐value_DF_	*p*‐Value	*N*	Estimate ± SE	*F*‐value_DF_	*p*‐Value	*N*	Estimate ± SE	*F*‐value_DF_	*p*‐Value	*N*	Estimate ± SE	*F*‐value_DF_	*p*‐value
Intercept	264	0.17 ± 0.2			293	−0.04 ± 0.15			240	0.12 ± 0.2			259	−0.01 ± 0.2		
Provisioning treatment (*Provisioned)*		0.01 ± 0.27	0.23_1,20.6_	.64		0.06 ± 0.21	0.49_1,9.31_	.50		−0.06 ± 0.27	0.01_1,20.6_	.91		−0.07 ± 0.27	0.23_1,23.8_	.63
Stage of reproduction			**3.79_2,237_**	.**02**			0.03_2,268_	.96			0.72_2,220_	.48			0.39_2,237_	.67
*(Pregnancy)*		−0.19 ± 0.2				0.04 ± 0.21				−0.33 ± 0.22				0.03 ± 0.21		
*(Lactation)*		0.24 ± 0.21				−0.06 ± 0.21				−0.03 ± 0.23				−0.05 ± 0.21		
Provisioning treatment × Stage of reproduction			0.31_2,237_	.73			0.11_2,271_	.89			0.8_2,220_	.45			0.83_2,237_	.43
*(Provisioned x Pregnancy)*		−0.10 ± 0.28				−0.01 ± 0.28				0.28 ± 0.3				−0.2 ± 0.28		
*(Provisioned x Lactation)*		−0.22 ± 0.29				0.11 ± 0.3				−0.08 ± 0.32				0.16 ± 0.3		

Note

Linear mixed model exploring the link between oxidative stress markers and provisioning treatment, stage of reproduction, and their interaction in pregnant females. *p*‐Values highlighted in bold do not remain significant after correction using the false discovery rate procedure.

**FIGURE 3:**
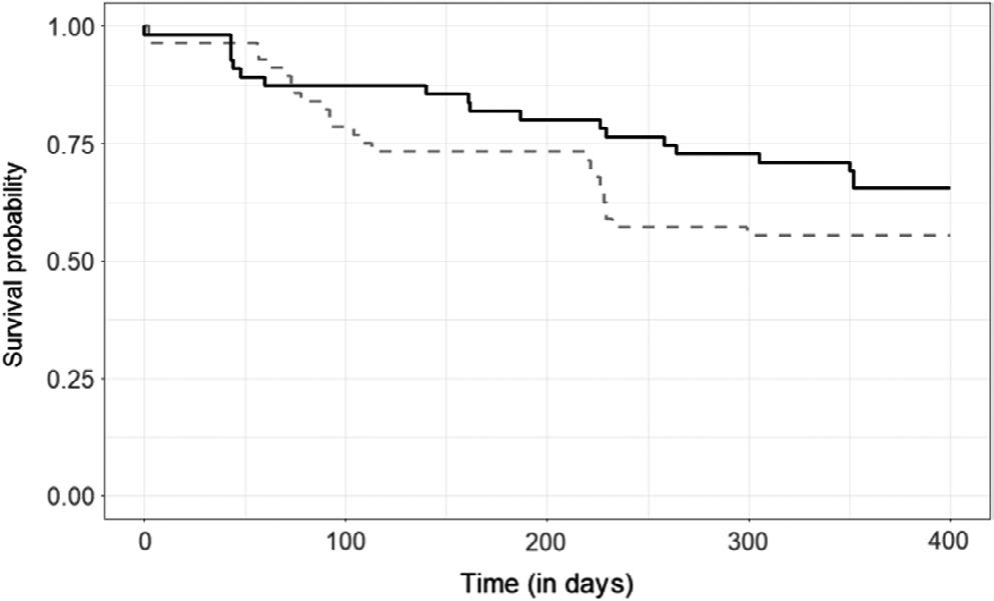
Survival of offspring from provisioned and non‐provisioned mothers. The black line represents offspring of provisioned mothers, while the grey dashed line represents offspring of non‐provisioned mothers

Maternal provisioning treatment did not significantly affect reproductive investment in terms of pre‐natal investment, or offspring body mass at emergence while controlling for the age at measurement (Table 2). Moreover, we did not find a significant effect of maternal provisioning treatment on offspring survival. The number of offspring emerging from the den was predicted by fetus number similarly in both provisioning treatments, while the effect of maternal provisioning treatment on offspring survival to 12 months was not statistically significant (Table 2, Figure 3).

**TABLE 2:** Test of prediction 1.2

	Pre‐natal investment				Offspring's body mass at emergence				Survival to emergence				Survival to 12 months			
	*N*	Estimate ± SE	*F*‐value _DF_	*p*‐Value	*N*	Estimate ± SE	*F*‐value _DF_	*p*‐Value	*N*	Estimate ± SE	*F*‐value _DF_	*p*‐Value	*N*	Estimate ± SE	Chi‐sq	*p*‐Value
Intercept	24	0.37 ± 0.06			92	146.03 ± 52.88			30	0.36 ± 0.66			108			
Offspring age at emergence		–	–	–		2.89 ± 1.15	**6.24 _1,56.3_**	.**01**		–	–	–		–	–	–
Fetus number		–	–	–		–	–	–		0.54 ± 0.23	**5.46 _1,26_**	.**03**		–	–	–
Provisioning treatment *(Provisioned)*		0.04 ± 0.05	0.69 _1,14.3_	.42		10.22 ± 14.9	0.46 _1,11.6_	.08		0.29 ± 1.13	0.07 _1,26_	.8		−0.28 ± 0.36	0.58	.44
Provisioning treatment × Fetus number *(Provisioned x Fetus number)*		–	–	–		–	–	–		−0.3 ± 0.4	0.58 _1,26_	.45		–	–	–

Note

Linear mixed model exploring the link between maternal investment/offspring survival and provisioning treatment, stage of reproduction, and their interaction in pregnant females. *p*‐values highlighted in bold do not remain significant after correction using the false discovery rate procedure.

### 3.2 How does oxidative stress shape reproduction?

**FIGURE 4:**
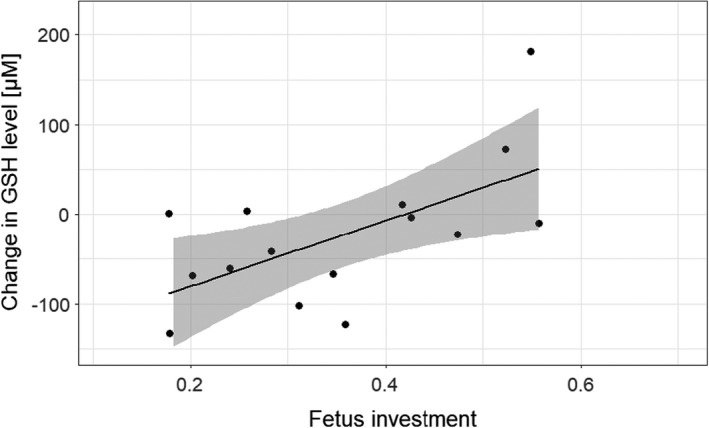
Relationship between within‐individual changes in GSH levels and pre‐natal investment. The line represents the regression line ±95% confidence interval (shaded region) with points representing the raw data

We investigated the oxidative cost hypothesis. Females that invested more in fetus production showed an increase in GSH levels during the breeding event (Table 3, Figure 4). However, intra‐individual changes in levels of other markers of oxidative stress were not significantly predicted by pre‐natal investment or offspring body mass at emergence (Table 3).

**TABLE 3:** Test of the oxidative cost hypothesis: prediction 2.1

	Change in PC				Change in MDA				Change in SOD				Change in GSH			
	*N*	Estimate ± SE	*F*‐value _DF_	*p*‐Value	*N*	Estimate ± SE	*F*‐value _DF_	*p*‐Value	*N*	Estimate ± SE	*F*‐value _DF_	*p*‐Value	*N*	Estimate ± SE	*F*‐value _DF_	*p*‐Value
*Model 1*	18				18				16				18			
Intercept		−0.48 ± 0.02				−0.08 ± 0.34				0.05 ± 0.52				−0.17 ± 0.2		
Pre‐natal investment		0.24 ± 0.24	0.96 _1,16_	.34		0.51 ± 0.27	3.59 _1,13.4_	.08		0.42 ± 0.40	1.09 _1,13.9_	.31		**0.53** ± **0.26**	**4.25 _1,16_**	.**05**
*Model 2*	42				42				40				38			
Intercept		−0.3 ± 0.3				0.15 ± 0.3				−0.08 ± 0.3				−0.02 ± 0.31		
Offspring body mass at emergence corrected		0.05 ± 0.16	0.11 _1,29.8_	.74		0.08 ± 0.11	0.48 _1,28.5_	.49		−0.02 ± 0.06	0.13 _1,26.6_	.72		0.04 ± 0.07	0.37 _1,21.7_	.55

Note

Linear mixed model exploring the link intra‐individual changes in oxidative status and reproductive investment. *p*‐Values highlighted in bold do not remain significant after correction using the false discovery rate procedure.

We then explored the oxidative constraint hypothesis. Neither pre‐natal investment nor offspring body mass at emergence was significantly predicted by any oxidative stress marker (Table 4).

**TABLE 4:** Test of the oxidative constraint hypothesis: prediction 2.2

	Pre‐natal investment				Offspring's body mass at emergence			
	*N*	Estimate ± SE	*F*‐value _DF_	*p*‐Value	*N*	Estimate ± SE	*F*‐value_DF_	*p*‐Value
Intercept	16	0.36 ± 0.05			63	96.8 ± 66.4		
Offspring age at emergence		–	–	–		**3.86** ± **1.5**	**6.65_1,51.7_**	.**01**
PC		0.07 ± 0.03	4.5_1,7.4_	.07		−1.27 ± 7.27	0.03_1,41.1_	.86
MDA		−0.01 ± 0.04	0.06_1,10.7_	.8		5.83 ± 6.31	0.85_1,54.6_	.36
SOD		0.01 ± 0.02	0.2_1,7.37_	.66		**15.82** ± **6.95**	**5.18_1,43.8_**	.**03**
GSH		**0.06** ± **0.02**	**6.67_1,7.32_**	.**03**		3.31 ± 6.72	0.24_1,55.9_	.62

Note

Models exploring the link between maternal investment and oxidative stress markers before reproduction, with the relevant covariates. *p*‐Values highlighted in bold do not remain significant after correction using the false discovery rate procedure.

**FIGURE 5:**
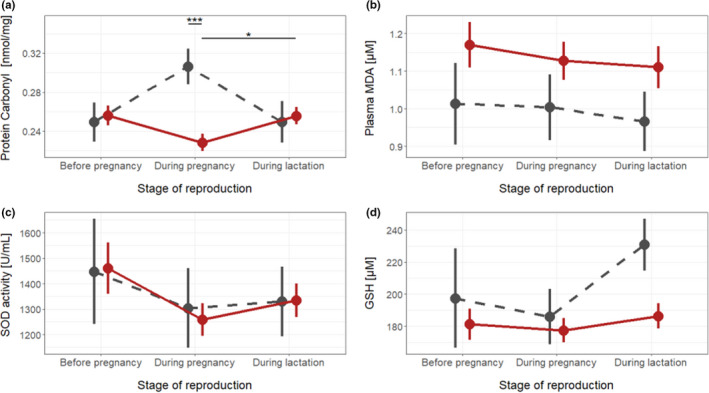
Dynamics of oxidative stress markers during the breeding event. (a) Protein Carbonyl level, (b) MDA level, (c) SOD activity, and (d) Glutathione level. Red dots and solid lines represent breeding females, while grey dots and dashed lines represent non‐breeding females. Symbols represent raw data means ± SE. Stars indicate statistical significance: ***: *p*‐value < .001, *: *p*‐value = .05

Finally, we examined the oxidative shielding hypothesis. First, we considered changes in oxidative stress markers over the course of reproduction in breeders compared to non‐breeders. Protein carbonyl levels varied in relation to the interaction between breeding status and stage of reproduction. Post hoc tests showed that protein carbonyl levels were similar in breeders and non‐breeders before the breeding event, but differed during pregnancy, with breeders exhibiting significantly lower levels of protein carbonyl compared to non‐breeders (*T*‐ratio = 3.58, *p*‐value < .001). Breeders showed an increase in protein carbonyl levels during lactation compared to levels during pregnancy (*T*‐ratio = −2.28, *p*‐value = .05) (Table 5, Figure 5). MDA, SOD, and GSH did not differ significantly in relation to the stage of reproduction, the breeding status, or their interaction (Table 5, Figure 5).

**TABLE 5:** Test of the shielding hypothesis: prediction 2.3.1

	Protein Carbonyl (nmol/mg protein)				MDA(μM)				SOD (U/ml)				GSH (μM)			
	*N*	Estimate ± SE	*F*‐value _DF_	*p*‐value	*N*	Estimate ± SE	*F*‐value_DF_	*p*‐Value	*N*	Estimate ± *SE*	*F*‐value _DF_	*p*‐Value	*N*	Estimate ± SE	*F*‐value _DF_	*p*‐Value
Intercept	313	0.17 ± 0.26			357	−0.02 ± 0.24			295	0.07 ± 0.25			313	−0.09 ± 0.26		
Stage of reproduction			1.01 _2,292_	.36			0.07 _2,324_	.93			0.7 _2,269_	.5			1.11 _2,285_	.33
*(Pregnancy)*		0.45 ± 0.31				−0.14 ± 0.29				−0.16 ± 0.31				−0.1 ± 0.3		
*(Lactation)*		−0.22 ± 0.32				−0.23 ± 0.32				−0.10 ± 0.32				0.34 ± 0.33		
Breeding status *(Breeding)*		−0.06 ± 0.26	2.87 _1,307_	.09		0.04 ± 0.25	1.46 _1,337_	.23		−0.02 ± 0.26	<0.01 _1,288_	.95		<0.01 ± 0.26	0.34 _1,306_	.56
Breeding status x Stage of reproduction			**4.88 _2,288_**	**<.01**			0.24 _2,319_	.79			<0.01 _2,271_	.99			0.54 _2,286_	.58
*(Breeding x Pregnancy)*		−0.73 ± 0.34				0.17 ± 0.32				0.01 ± 0.34				0.006 ± 0.33		
*(Breeding x Lactation)*		0.27 ± 0.36				0.23 ± 0.35				0.04 ± 0.36				−0.31 ± 0.36		

Note

Linear mixed model exploring the link between oxidative stress markers and stage of reproduction, breeding status, and their interaction. *p*‐Values highlighted in bold do not remain significant after correction using the false discovery rate procedure.

**FIGURE 6:**
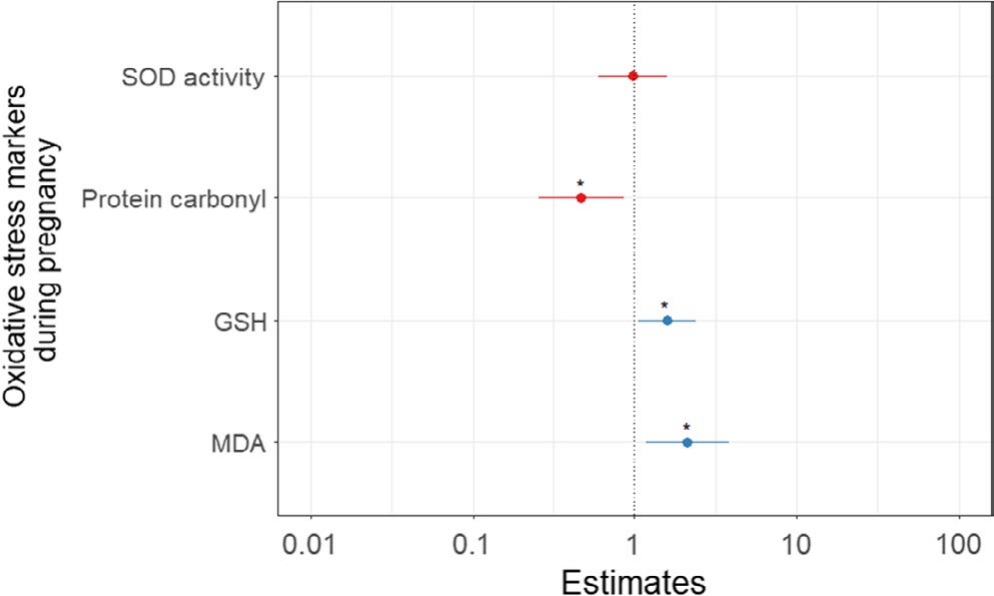
Correlation between survival to 12 months and maternal oxidative stress markers measured during pregnancy. Hazard ratios are shown. * Indicates statistical significance. Blue indicates estimates greater than 1, red indicates estimates equal or below to 1

Second, we checked whether oxidative stress levels during pregnancy influenced maternal investment and offspring survival. Pre‐natal investment, offspring body mass at emergence, and survival to emergence were not significantly impacted by maternal levels of oxidative stress during pregnancy (Table 6). Interestingly, survival to 12 months was negatively correlated with maternal levels of protein carbonyls during pregnancy, while positively correlated with both GSH and MDA (Table 6, Figure 6).

**TABLE 6:** Test of the shielding hypothesis: prediction 2.3.2

	Pre‐natal investment				Offspring body mass at emergence				Survival to emergence				Survival to 12 months			
	*N*	Estimate ± SE	*F*‐value _DF_	*p*‐value	*N*	Estimate ± SE	*F*‐value_DF_	*p*‐Value	*N*	Estimate ± SE	*F*‐value _DF_	*p*‐Value	*N*	Estimate ± SE	Chi‐sq	*p*‐Value
Intercept	21	0.39 ± 0.05			50	190.4 ± 69.72			26	0.42 ± 0.6			56	–	–	–
Offspring age at emergence		–	–	–		1.93 ± 1.62	1.42 _1,35.3_	.24		–	–	–		–	–	–
Number of fetuses		–	–	–		–	–	–		0.38 ± 0.22	3.07 _1,19.9_	.09		–	–	–
PC		0.01 ± 0.04	0.14 _1,14_	.71		8.45 ± 9.59	0.77 _1,39_	.38		−0.12 ± 0.17	0.52 _1,19_	.48		−**0.75** ± **0.31**	**6.01**	.**01**
MDA		0.03 ± 0.03	0.83 _1,15.7_	.37		−9.93 ± 9.35	1.13 _1,18.5_	.30		−0.18 ± 0.17	1.12 _1,11.1_	.31		**0.75** ± **0.3**	**6.39**	.**01**
SOD		−<0.01 ± 0.03	<0.01 _1,15.2_	.99		11.52 ± 8.83	1.7 _1,11.6_	.22		0.11 ± 0.16	0.46 _1,6.5_	.52		−0.02 ± 0.25	0.006	.93
GSH		0.05 ± 0.02	4.12 _1,9.3_	.07		3.06 ± 9.50	0.10 _1,16.7_	.75		0.20 ± 0.15	1.6 _1,8.7_	.23		**0.46** ± **0.21**	**4.99**	.**02**

Note

Models exploring the link between maternal investment as well as offspring survival and oxidative stress markers during pregnancy, with the relevant covariates. *p*‐Values highlighted in bold do not remain significant after correction using the false discovery rate procedure.

Finally, we checked whether within‐individual changes in oxidative stress markers were associated with baseline levels prior to reproduction. After adjusting the values of the within‐individual changes in oxidative stress markers for regression toward the mean (Kelly & Price, 2005), we found that intra‐individual changes were not predicted by baseline levels prior to reproduction (PC: estimate ± SE = −0.09 ± 0.17, *F*‐value _1,38.97_ = 0.28, *p*‐value = .6; MDA: estimate ± SE = 0.02 ± 0.16, *F*‐value_1,39.16_ = 0.02, *p*‐value = .89; SOD: Estimate ± SE = −0.15 ± 0.15, *F*‐value_1,34.83_ = 1.07, *p*‐value = .31; and GSH: estimate ± SE = −0.006 ± 0.15, *F*‐value_1,34.87_ < 0.01, *p*‐value = .97). However, the intra‐individual change in glutathione level was positively correlated with body mass before pregnancy (GSH: estimate ± SE = 0.4 ± 0.15, *F*‐value_1,32.17_ = 7.44, *p*‐value = .01), with heavier females displaying a stronger increase in glutathione levels during pregnancy compared to lighter females. None of the intra‐individual changes in other markers were linked to body mass during pregnancy (PC: estimate ± SE = 0.22 ± 0.15, *F*‐value_1,31.16_ = 2.18, *p*‐value = .15; MDA: estimate ± SE = −0.04 ± 0.15, *F*‐value_1,19.53_ = 0.07, *p*‐value = .79; and SOD: Estimate ± SE = −0.25 ± 0.13, *F*‐value_1,21.24_ = 3.17, *p*‐value = .09).

## 4 DISCUSSION

We explored how oxidative stress can shape reproduction, by testing three mechanisms: oxidative cost, oxidative constraint, and oxidative shielding. Using a long‐term provisioning experiment, we also tested the linkages among maternal nutrition, oxidative stress, and offspring production. Eggs of domestic chickens were used for the experimental provisioning, as bird eggs make up part of the natural diet of banded mongooses (Hinton & Dunn, 1967). As eggs are rich in antioxidants including vitamin E (Nimalaratne & Wu, 2015; Seuss‐baum, 2007), we predicted that experimental provisioning would improve individuals’ oxidative state, either by allowing increased endogenous antioxidant production, and/or by providing exogenous antioxidants. However, in contrast to previous studies (Fletcher et al., 2013; Giordano et al., 2015), we found no significant effect of dietary provisioning of females on their oxidative state. Moreover, provisioning did not affect offspring production by females. What then did provisioned mothers do with the extra resources that they received? It is unlikely that our experimental provisioning represented a negligible input to the diet, as one egg represents about one third of daily energy requirements (Laver et al., 2020). Moreover, both provisioned and non‐provisioned females increased their body mass after the start of the experiment, above and beyond the effect of age (see Figure S3). This result suggests that the experimental provisioning was successful. It is possible that fed individuals subsequently reduced their foraging effort, thus leading to similar nutrition in provisioned and non‐provisioned individuals, but that would not explain why all females became heavier. Alternatively, non‐provisioned individuals could have increased their foraging effort to exhibit competitive growth, whereby they would increase their body mass to remain competitive with provisioned females as shown recently in meerkats (*Suricata suricatta*) and anemone clownfish (*Amphiprion percula*) (Huchard et al., 2016; Reed et al., 2019). Finally, natural food availability could have been relatively high during the experiment, thus obscuring any effect of provisioning, as shown in a meta‐analysis based on studies of birds (Ruffino et al., 2014). Indeed, mongoose population density was extremely low at the beginning of the experimental period, and increased slowly, which likely conferred unusually profitable foraging opportunities. Specifically, this might have meant that females were not limited in terms of antioxidant defenses, either because they were able to acquire sufficient dietary antioxidants during foraging, or they had enough resources to synthesize endogenous antioxidants (Beaulieu & Schaefer, 2013). Further work is required to understand what mechanisms may be responsible for the results observed. In general, the effect of provisioning on wild animals is poorly understood. While food supplementation in wild animals often leads to a decrease in home range size, an increase in body mass, and advances the date of first breeding, it has limited impact on offspring production (Boutin, 1990). Similar results were found in a study of effects of feeding on anthropogenic food waste by banded mongooses; individuals that fed on refuse were heavier and carried more fetuses (Otali & Gilchrist, 2004). However, despite these apparent beneficial effects, conception rate, number of emerging offspring, and survival to 3 months of age were similar in refuse feeding and non‐refuse feeding groups, suggesting that extra nutritional resources do not necessarily lead to increased reproductive success in banded mongooses (Otali & Gilchrist, 2004).

Our results provide evidence that oxidative stress can shape reproduction in multiple ways. First, we found some limited evidence for the “oxidative cost” hypothesis, as increased investment in reproduction was associated with increased levels of the antioxidant glutathione. Such increase in antioxidant defenses associated with stable levels of oxidative damage suggests an upregulation of antioxidant defenses in response to an oxidative challenge, to prevent an increase in oxidative damage (Beaulieu & Costantini, 2014; Costantini & Verhulst, 2009; Hõrak & Cohen, 2010). A similar pattern was reported in zebra finches after an oxidative challenge induced by diquat dibromide, which elicited an increase in antioxidant capacity, while it did not affect damage levels (Tomášek et al., 2016). Thus, our results suggest that increased offspring production is likely to pose an oxidative challenge. It is perhaps not surprising that we found only limited evidence for an oxidative cost of reproduction, as individuals may have adjusted offspring production to their own condition.

We found no support for the “oxidative constraint” hypothesis. Similarly, Viblanc et al. (2018) found no support for that hypothesis, as females Columbian ground squirrels that displayed higher levels of oxidative stress before pregnancy produced larger litters at birth. These results are in contrast to the pre‐existing empirical evidence that supports the oxidative constraint hypothesis (Costantini et al., 2016; Montoya et al., 2016; Stier et al., 2012). It would suggest that in banded mongooses and in Columbian ground squirrels, constraining investment in reproduction is not the primary mechanism used by breeders to maintain low levels of oxidative stress.

Finally, consistent with the “oxidative shielding” hypothesis, plasma levels of protein carbonyls were lower in pregnant females compared to non‐breeders. Maternal levels of protein carbonyls during pregnancy were also negatively correlated with offspring survival to 1 year of age. This suggests that protein carbonyls may have detrimental consequences that can transmit across generations. A similar negative effect of protein carbonyls on survival has been reported in Soay sheep (*Ovis aries*), where male lambs with higher plasma levels of protein carbonyls had lower survival to the first winter (Christensen et al., 2015). Moreover, we found that maternal levels of the antioxidant glutathione correlated positively with offspring survival to 1 year of age, again suggesting long‐term intergenerational consequences of maternal oxidative state. Although the mechanisms underlying such long‐term effects of oxidative stress markers on longevity are unknown, we can hypothesize a few ways such effects may arise. Pre‐natal exposure to oxidative stress could contribute to oxidative damage directly, via damage transfer from the mother to its offspring (see Supplementary Material for relationship between offspring and maternal oxidative stress markers). However, it could also have indirect effects by impacting the ability of the physiological systems to cope with oxidative stress over the long term (Isaksson et al., 2011), by altering signal transduction, or more generally by modifying gene expression via epigenetic changes modulated by oxidative stress levels (García‐Guede et al., 2020; Hitchler & Domann, 2007). Specifically, glutathione is proposed to directly influence epigenetic mechanisms via its role on S‐adenosylmethionine, a cofactor used by the epigenetic machinery (Hitchler & Domann, 2007). Surprisingly, levels of oxidative lipid damage (MDA) in pregnant females were also positively correlated with offspring survival to 1 year of age. However, levels of MDA, glutathione, and protein carbonyls were not significantly correlated. This pattern is opposite to what was previously found in banded mongooses, where maternal MDA levels correlated negatively with mixed‐maternity litter survival (Vitikainen et al., 2016). However, in Vitikainen et al.’s (2016) study, levels of MDA during pregnancy were markedly and significantly higher than the present study (Vitikainen et al., 2016: mean ± SE = 1.74 ± 0.05; this study: mean ± SE = 1.11 ± 0.03; *T*‐value = 9.16, *p*‐value < .001). Negative impacts of oxidative stress may not arise at relatively low levels of lipid peroxidation.

Interestingly, our data suggest that oxidative costs can be observed in terms of variation in antioxidant levels, while oxidative damage levels may remain stable, suggesting that individuals were mostly able to mitigate against increased risk of oxidative stress in association with reproduction. Additionally, we report for the first time a decrease in protein carbonyl levels during pregnancy. Indeed, earlier work has reported elevated protein carbonyl levels during breeding in banded mongooses (Vitikainen et al., 2016) and Brandt's voles *(Lasiopodomys brandtii)* (Xu et al., 2014), and as a result of meta‐analysis (Blount et al., 2016). This suggests that maintaining low levels of protein carbonyls during reproduction might rarely be possible, perhaps only where individuals are in good condition and experiencing relatively low levels of oxidative stress. Together with our finding that MDA levels were low and positively correlated with offspring survival, these results suggest that environmental conditions were rather benign during the experiment, thus allowing individuals to maintain low levels of oxidative stress, even during reproduction.

The change in circulating levels of oxidative stress markers during breeding appeared to be independent of levels measured prior to reproduction. Such absence of linkage suggests that, contrary to our prediction, individuals do not adjust levels of oxidative stress markers during breeding in relation to their baseline levels. However, we found that heavier females before pregnancy displayed a higher increase in glutathione levels during pregnancy compared to lighter individuals. Potentially, this could suggest that a tailored mitigation might be too costly for individuals of lower quality, which might be expected to display relatively high baseline levels of oxidative stress. For example, it was shown in house sparrows (*Passer domesticus*) that individuals at the bottom of the dominance hierarchy displayed more oxidized ejaculates compared to those higher in the hierarchy, suggesting that restricted access to resources prevented individuals from protecting their ejaculates from oxidative stress (Rojas Mora et al., 2017).

Longitudinal sampling across the annual calendar and including breeding events can give powerful insights into how oxidative stress may shape reproduction. Indeed, it is now well established that breeding individuals often vary considerably in baseline levels of oxidative stress (Alajbeg et al., 2017; Bodey et al., 2020; Herborn et al., 2011; Martinez‐Moral & Kannan, 2019). Within‐individual changes in oxidative stress levels associated with reproductive effort can therefore be more informative than standalone measurements. Longitudinal data are also essential for testing the oxidative shielding hypothesis, which predicts decreased oxidative damage levels during stages of reproduction when offspring are physiologically dependent on their mothers. The present study illustrates the value of combining different sampling time points, including single time point measurements and intra‐individual changes in order to investigate how oxidative stress may shape reproduction (see also Viblanc et al., 2018).

In conclusion, our results support the view that oxidative stress is an important factor that shapes reproduction. Oxidative stress is likely to act as a cost of reproduction, with intergenerational consequences. As such, oxidative stress may represent not only a proximate cost of reproduction but also an ultimate cost of reproduction. Fitness may be enhanced where individuals are able to avoid high levels of oxidative damage when breeding, either by tailoring their investment in reproduction to their baseline oxidative state, and/or by lowering levels of damage and increasing antioxidant protection during reproduction. In banded mongooses, it appears that oxidative constraint and oxidative shielding are likely to be alternative mechanisms by which oxidative stress can shape reproduction in order to optimize an individual's life reproductive success.

## CONFLICT OF INTEREST

The authors have no competing interests.

## AUTHOR CONTRIBUTIONS

**Magali Meniri:** Data curation (equal); Formal analysis (equal); Investigation (equal); Methodology (equal); Writing – original draft (equal). **Elsa Evans:** Data curation (equal); Investigation (equal); Writing – review & editing (equal). **Faye J. Thompson:** Data curation (equal); Investigation (equal); Writing – review & editing (equal). **Harry H. Marshall:** Data curation (equal); Investigation (equal); Writing – review & editing (equal). **Hazel J. Nichols:** Data curation (equal); Investigation (equal); Methodology (equal); Writing – review & editing (equal). **Gina Lewis:** Investigation (equal); Writing – review & editing (equal). **Lauren Holt:** Investigation (equal); Methodology (equal); Writing – review & editing (equal). **Emma Davey:** Investigation (equal); Methodology (equal); Writing – review & editing (equal). **Christopher Mitchell:** Investigation (equal); Methodology (equal); Writing – review & editing (equal). **Rufus A. Johnstone:** Conceptualization (equal); Funding acquisition (equal); Investigation (equal); Resources (equal); Supervision (equal); Writing – review & editing (equal). **Michael A. Cant:** Conceptualization (equal); Funding acquisition (equal); Investigation (equal); Resources (equal); Supervision (equal); Writing – review & editing (equal). **Jonathan D. Blount:** Conceptualization (equal); Funding acquisition (equal); Investigation (equal); Methodology (equal); Project administration (equal); Resources (equal); Supervision (equal); Writing – original draft (supporting).

### OPEN RESEARCH BADGES

This article has been awarded Open Data, Open Materials Badges. All materials and data are publicly accessible via the Open Science Framework and provided as part of the Supplementary Material.

## Supporting information

Supplementary Material

## Data Availability

The data that support the findings of this study are deposited in the Dryad repository: https://doi.org/10.5061/dryad.wdbrv15qs.
