# Corrigendum

**DOI:** 10.1002/ece3.9466

**Published:** 2022-11-05

In the recent article by Meniri et al. (2022), the survival effect was interpreted the wrong way around, with a positive effect on survival interpreted as a negative one in Figure 6. The author would like to amend the result section and the associated paragraph in the discussion where they interpret this result.

In the Abstract section, in the penultimate final sentence, the word “negatively” was removed. While in the last sentence of the fourth paragraph of Section 3.2, the words “negatively” and “positively” have been replaced with “positively” and “negatively” respectively.

The fourth paragraph from the “Discussion” section is shown below:

Finally, we found partial support for the “oxidative shielding” hypothesis. Indeed, consistent with this idea, plasma levels of protein carbonyls were lower in pregnant females compared with nonbreeders. Moreover, regarding the long‐term impact of maternal oxidative stress markers on offspring survival, we found that levels of oxidative lipid damage (MDA) in pregnant females were negatively correlated with offspring survival to 1 year of age, as predicted by the oxidative shielding hypothesis and previously found in the same species (Vitikainen et al., 2016). However, contrary to our predictions, maternal levels of protein carbonyls during pregnancy were positively correlated with offspring survival to 1 year of age. That is, offspring whose mothers had higher levels of protein carbonyls exhibited higher early‐life survival. In addition, we found that maternal levels of the antioxidant glutathione correlated negatively with offspring survival to 1 year of age, a result opposite to our predictions. These results could arise if mothers of better “quality” could tolerate higher levels of damage, without negatively impacting their offspring, while producing higher “quality” offspring that survived better (Reznick et al., 2000). Indeed, high levels of protein carbonyls in pregnant mothers could be a signature of high protein turnover and high investment in fetal protein synthesis, which would benefit offspring (Blount et al., 2016). Alternatively, we cannot exclude the possibility that maternal oxidative shielding comes at a cost to offspring, while benefitting the mother. However, it appears more likely, if mothers are limited in resources for that breeding event, to lower offspring production and thus the level of oxidative cost, rather than having their offspring bear the cost of oxidative shielding. Indeed, oxidative shielding should be beneficial to the offspring in many ways (Blount et al., 2016). Overall, maternal oxidative state appears to have long‐term, albeit complex, intergenerational consequences. Although the mechanisms underlying such long‐term effects of maternal oxidative stress markers on offspring survival are unknown, we can hypothesize a few ways such effects may arise. Prenatal exposure to oxidative stress could contribute to oxidative damage directly, via damage transfer from the mother to its offspring (see Supplementary material for the relationship between offspring and maternal oxidative stress markers). However, it could also have indirect effects by impacting the ability of physiological systems to cope with oxidative stress over the long term (Isaksson et al., 2011), by altering signal transduction, or more generally by modifying gene expression via epigenetic changes modulated by oxidative stress levels (García‐Guede et al., 2020; Hitchler & Domann, 2007).

The fifth paragraph from the Discussion section was entirely removed.

These have been corrected in the online version.

The authors apologize for these errors.
