# Supplementary material for: Untangling the oxidative cost of reproduction: An analysis in wild banded mongooses
Source: Ecol Evol. 2022 Mar 8;12(3):e8644. doi: 10.1002/ece3.8644 (PMC8928901; doi:10.1002/ece3.8644)
Supplement: Supplementary file 1 — Supplementary Material [file ECE3-12-e8644-s001.doc]

**Supplementary material**

**Untangling the oxidative cost of reproduction: an experimental test in banded mongooses**

**Ultrasound scanning**

**
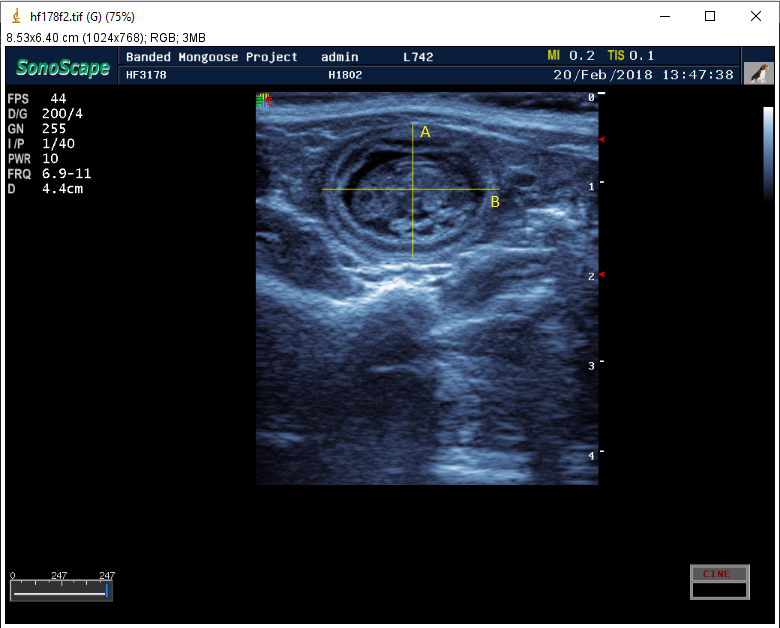
**

Figure S1: Foetal ultrasound image. The elliptical shape measured using yellow perpendicular lines ‘A’ and ‘B’ makes up the gestational sac. Measurements ‘A’ and ‘B’ correspond to the terms in the formula for cross-sectional area.

**Quantification of malondialdehyde (MDA)**

Plasma malondialdehyde was determined using high performance liquid chromatography (HPLC) with fluorescence detection following (Nussey et al. 2009) with some modifications described here. All chemicals were HPLC grade, and chemical solutions were prepared using ultra-pure water (Milli-Q Synthesis; Millipore, Watford, UK). Briefly, 20 µl of plasma or standard (1,1,3,3-tetraethoxypropane, TEP; see below), 20 µl 0.05% (w/v) butylated hydroxytoluene solution in 95% ethanol, and 160 µl of 0.44M phosphoric acid solution were added to a 2ml screw cap reaction tube. To initiate the reaction, 40 µl of 42mM 2- thiobarbituric acid (TBA) was added. Tubes were then capped and briefly vortexed, before being incubated on a dry heat block for 1 hour at 100°C to allow formation of MDA-TBA adducts. After the incubation period, samples were placed on ice for five minutes to stop any further reaction. nButanol (160 µl) was added, and tubes were vortexed for 20 seconds. Samples were then centrifuged at 12,000 x g for 3 min at 4°C. A 100 µl aliquot of the upper butanol phase was carefully transferred to a 0.3ml crimp top HPLC vial. Samples (40 µl) were injected into an Agilent 1200 series HPLC system (Agilent Technologies, California, USA) fitted with Thermo Scientific Hypersil 5µ ODS 100 x 4.6mm column (Patr no. 30105-104630) The mobile phase was methanol-buffer (40:60, v/v), the buffer being a 50mM anhydrous solution of potassium monobasic phosphate at pH 6.8 (adjusted using 5M potassium hydroxide solution), running isocratically over 3.5 min at a flow rate of 1 ml min-1 . The column oven was set at 37°C. Peaks were collected using a fluorescence detector (Agilent G1321C), with excitation and emission wavelengths of 515nm and 553nm, respectively. Peaks were quantified relative to an external calibration curve prepared using a TEP stock solution (5µM in 40% ethanol) serially diluted using 40% ethanol to give known values in the range 0 – 5 µM.

**Quantification of protein carbonyls**

Quantification is based on the reaction between DNPH (2,4-dinitrophenylhydrazine) and protein carbonyls forming a protein hydrozone, measured using a plate reader (Spectramax M2; Molecular Devices, USA). The carbonyl content is standardized to protein concentration of the sample. Due to limitations in the amount of plasma available as well as the high protein content of samples, 50 μl of plasma was used in the sample and control tubes instead of the 200 μl recommended in the kit instructions. 50 μl of sample was added to each sample and control tube. 200 μl of DNPH to was added to sample tubes and 200μl of 2.5M HCl to control tubes, and incubated in the dark for 1 hour. Samples were vortexed every 15 mins during the incubation period. 125 μl of 50% TCA was added to each tube and vortexed. Tubes were incubated on ice for 15 mins and centrifuged at 12 g for 15mins at 4C. To wash the pellet, the supernatant was drawn off, 500 μl of 1:1 solution of Ethanol : Ethyl acetate was added to each tube, the pellet was broken up and all tubes were vortexed and centrifuged at 12 g for 15mins at 4C. The previous washing steps of the pellets were repeated once more. The supernatant was drawn off a final time and pellets were resuspended in 500μl of a Guanidine hydrochloride solution. All tubes were vortexed and centrifuged at 10g for 10 mins at 4C. 220 μl of supernatant for the samples and control tubes were transferred in duplicates on 96 wells plate and the absorbance was measured at 370 nm. A Bradford assay was performed to determine the protein concentration of the pellets. For that, a standard curve was prepared using Bovine Serum Albumin 2mg/ml and a Guanidine hydrochloride solution. 200 μl of each standard was added in duplicate to a 96 wells plate, and 20 μl of control tubes were added to 180 μl of the Guanidine hydrochloride solution into control wells in duplicate. Absorbance was measured at 280nm. If a difference of more than 50% was observed between duplicates within-plate, the sample was re-run in a subsequent batch.

**Quantification of superoxide Dismutase activity (SOD)**

Quantification is based on the detection of superoxide radicals generated by xanthine oxidase and neutralized by SOD, using a plate reader (Spectramax M2; Molecular Devices, USA). One unit is defined as the amount of enzyme needed to exhibit 50% dismutation of the superoxide radical. RBC samples were diluted in a 1:10 w/v solution using ice cold H_2_O MQ, then centrifuged at 10 000 x g for 15 min at 4°C. The supernatant was collected and further diluted 1:100 with sample buffer for quantification. The assay was conducted according to kit instructions. If a difference of more than 50% was observed between duplicates within-plate, the sample was re-run in a subsequent batch.

**Quantification of glutathione (GSH)**

Quantification is based on the detection of 5-thio-2-nitrobenzoic acid (TNB), produced by a reaction between the sulfhydryl group of GSH and 5,5’-dithio-bis-2-nitrobenzoic acid (DNTB), using a plate reader (Spectramax M2; Molecular Devices, USA).

Similar to the SOD assay, RBC samples were diluted in a 1:10 w/v solution using ice cold H_2_O MQ, then centrifuged at 10 000 x g for 15 min at 4°C. The supernatant was collected and deproteinated before the assay. The assay was conducted according to kit instructions. If a difference of more than 50% was observed between duplicates within-plate, the sample was re-run in a subsequent batch.

**Controlling for regression to the mean**

To account for potential problems with regression to the mean, we calculated within-individual changes in levels of oxidative stress markers (PC, MDA, SOD or GSH) during the breeding event as an adjusted change between the marker levels during pregnancy and the levels before pregnancy using the formulae from Kelly & Price (2005):

Δ𝑋 = (𝑋_2_ −
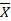
_2_) − 𝑝̂(𝑋_1_ −
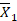
 )

and


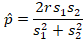


where 𝑋_1_,
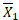
 and 𝑠_1_ are the oxidative stress markers’ level, the mean and standard deviation of the markers’ level before pregnancy, and 𝑋_2_,
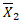
 and 𝑠_2_ are the markers’ level , the mean and standard deviation of the markers’ level during pregnancy, and 𝑟 is the correlation between the markers’ levels before and during pregnancy.

**Link between offspring oxidative stress levels and maternal oxidative stress levels**

To check whether offspring oxidative stress levels were related to maternal oxidative stress levels according to the treatment, we ran three sets of linear mixed models. Offspring oxidative stress levels were used as a response variable, and the corresponding maternal oxidative stress marker (PC, MDA, SOD and GSH) measured either before pregnancy, during pregnancy or during lactation were used as explanatory variables. Litter identity and maternal identity were included as random effects.

Table S1: Linear mixed model exploring the link between offspring levels of oxidative stress markers and maternal levels of oxidative stress markers, maternal provisioning treatment, and their interaction.

|  |  | **Offspring level of**  **Protein Carbonyl** (nmol/mg protein) | | | |  | **Offspring level of**  **MDA** (μM) | | | |  | **Offspring activity of**  **SOD** (U/ml) | | | |  | **Offspring level of**  **GSH** (μM) | | | |
| --- | --- | --- | --- | --- | --- | --- | --- | --- | --- | --- | --- | --- | --- | --- | --- | --- | --- | --- | --- | --- |
|  |  | N | Estimate  +/- se | F-value  _DF_ | P-value |  | N | Estimate  +/- se | F-value _DF_ | P-value |  | N | Estimate  +/- se | F-value _DF_ | P-value |  | N | Estimate  +/- se | F-value _DF_ | P-value |
|  |  |  |  |  |  |  |  |  |  |  |  |  |  |  |  |  |  |  |  |  |
| ***Model 1: Maternal levels before pregnancy*** |  |  |  |  |  |  |  |  |  |  |  |  |  |  |  |  |  |  |  |  |
| Intercept |  | 63 | 0.53+/-0.11 |  |  |  | 66 | 1.3+/-0.19 |  |  |  | 67 | 428.9+/-378.25 |  |  |  | 68 | 190.38+/-28.6 |  |  |
|  |  |  |  |  |  |  |  |  |  |  |  |  |  |  |  |  |  |  |  |  |
| **Marker’s level before pregnancy** |  |  | -0.7+/-0.37 | 1.52 _1,39.46_ | 0.23 |  |  | -0.16+/-0.16 | 0.01 _1,25.7_ | 0.9 |  |  | **0.55+/-0.23** | **4.92 _1,44.54_** | **0.03** |  |  | -0.08+/-0.15 | 0.01 _1,49_ | 0.91 |
|  |  |  |  |  |  |  |  |  |  |  |  |  |  |  |  |  |  |  |  |  |
| Provisioning treatment (*Provisioned)* |  |  | -0.29+/- 0.17 | 3.06 _1,29.56_ | 0.09 |  |  | -0.49+/-0.26 | 3.36 _1,46.57_ | 0.07 |  |  | 363.42+/-391.77 | 0.86 _1,56.74_ | 0.36 |  |  | -62.7+/-32.58 | 3.7 _1,61.05_ | 0.06 |
|  |  |  |  |  |  |  |  |  |  |  |  |  |  |  |  |  |  |  |  |  |
| Provisioning treatment *(Provisioned)* x  Marker’s level before pregnancy |  |  | 1.05 +/-0.57 | 3.43 _1,34.42_ | 0.07 |  |  | 0.43+/-0.24 | 3.23 _1,47.85_ | 0.08 |  |  | -0.25+/-0.25 | 1.02 _1,56.06_ | 0.31 |  |  | 0.19+/-0.17 | 1.25 _1,61.31_ | 0.27 |
|  |  |  |  |  |  |  |  |  |  |  |  |  |  |  |  |  |  |  |  |  |
|  |  |  |  |  |  |  |  |  |  |  |  |  |  |  |  |  |  |  |  |  |
|  |  |  |  |  |  |  |  |  |  |  |  |  |  |  |  |  |  |  |  |  |
| ***Model 2: Maternal levels during pregnancy*** |  |  |  |  |  |  |  |  |  |  |  |  |  |  |  |  |  |  |  |  |
|  |  |  |  |  |  |  |  |  |  |  |  |  |  |  |  |  |  |  |  |  |
| Intercept |  | 58 | 0.25+/-0.07 |  |  |  | 67 | 1.21+/-0.2 |  |  |  | 52 | 925.74+/-257.51 |  |  |  | 50 | 208.25+/-48.8 |  |  |
|  |  |  |  |  |  |  |  |  |  |  |  |  |  |  |  |  |  |  |  |  |
| Marker’s level during pregnancy |  |  | 0.29+/-0.26 | 1.39 _1,52.64_ | 0.24 |  |  | -0.08+/-0.17 | 0.29_1,57.7_ | 0.59 |  |  | 0.18+/-0.15 | 0.34 _1,35.9_ | 0.56 |  |  | -0.10+/-0.27 | 0.17 _1,27.18_ | 0.68 |
|  |  |  |  |  |  |  |  |  |  |  |  |  |  |  |  |  |  |  |  |  |
| Provisioning treatment (*Provisioned)* |  |  | 0.04+/- 0.12 | 0.11 _1,52.79_ | 0.74 |  |  | 0.10+/-0.28 | 0.12 _1,59.48_ | 0.72 |  |  | 229.3+/-261.38 | 0.77 _1,40.7_ | 0.38 |  |  | -81.85+/-52.4 | 2.44 _1,22.6_ | 0.13 |
|  |  |  |  |  |  |  |  |  |  |  |  |  |  |  |  |  |  |  |  |  |
|  |  |  |  |  |  |  |  |  |  |  |  |  |  |  |  |  |  |  |  |  |
| Provisioning treatment *(Provisioned)* x  Marker’s level during pregnancy |  |  | -0.09 +/-0.44 | 0.04 _1,52.85_ | 0.83 |  |  | 0.02+/-0.24 | 0.005 _1,61.58_ | 0.94 |  |  | -0.21+/-0.17 | 1.55 _1,40.17_ | 0.22 |  |  | 0.22+/-0.28 | 0.66 _1,23.28_ | 0.42 |
|  |  |  |  |  |  |  |  |  |  |  |  |  |  |  |  |  |  |  |  |  |
|  |  |  |  |  |  |  |  |  |  |  |  |  |  |  |  |  |  |  |  |  |
|  |  |  |  |  |  |  |  |  |  |  |  |  |  |  |  |  |  |  |  |  |
| ***Model 3: Maternal levels during lactation*** |  |  |  |  |  |  |  |  |  |  |  |  |  |  |  |  |  |  |  |  |
|  |  |  |  |  |  |  |  |  |  |  |  |  |  |  |  |  |  |  |  |  |
| Intercept |  | 68 | 0.34+/-0.06 |  |  |  | 76 | 1.43+/-0.21 |  |  |  | 71 | 689.19+/-462.39 |  |  |  | 70 | 187.22+/-34.95 |  |  |
|  |  |  |  |  |  |  |  |  |  |  |  |  |  |  |  |  |  |  |  |  |
| Marker’s level during lactation |  |  | -0.06+/-0.18 | 0.26 _1,63.47_ | 0.61 |  |  | -0.2+/-0.16 | 1.64_,25.3_ | 0.21 |  |  | 0.36+/-0.32 | 0.16 _1,41.36_ | 0.69 |  |  | -0.17+/-0.18 | 2.74 _1,42.44_ | 0.10 |
|  |  |  |  |  |  |  |  |  |  |  |  |  |  |  |  |  |  |  |  |  |
| Provisioning treatment (*Provisioned)* |  |  | 0.03+/- 0.11 | 0.10 _1,63.56_ | 0.75 |  |  | -0.05+/-0.25 | 0.04 _1,71.5_ | 0.84 |  |  | 703.31+/-548.64 | 1.64 _1,20.17_ | 0.21 |  |  | **-98.97+/-40.77** | **5.89 _1,46.7_** | **0.02** |
|  |  |  |  |  |  |  |  |  |  |  |  |  |  |  |  |  |  |  |  |  |
| **Provisioning treatment *(Provisioned)* x**  **Marker’s level during lactation** |  |  | -0.10 +/-0.36 | 0.08 _1,63.14_ | 0.78 |  |  | 0.05+/-0.20 | 0.05 _1,71.7_ | 0.81 |  |  | -0.48+/-0.37 | 1.66 _1,34.88_ | 0.21 |  |  | **0.53+/-0.21** | **6.41 _1,60.2_** | **0.01** |


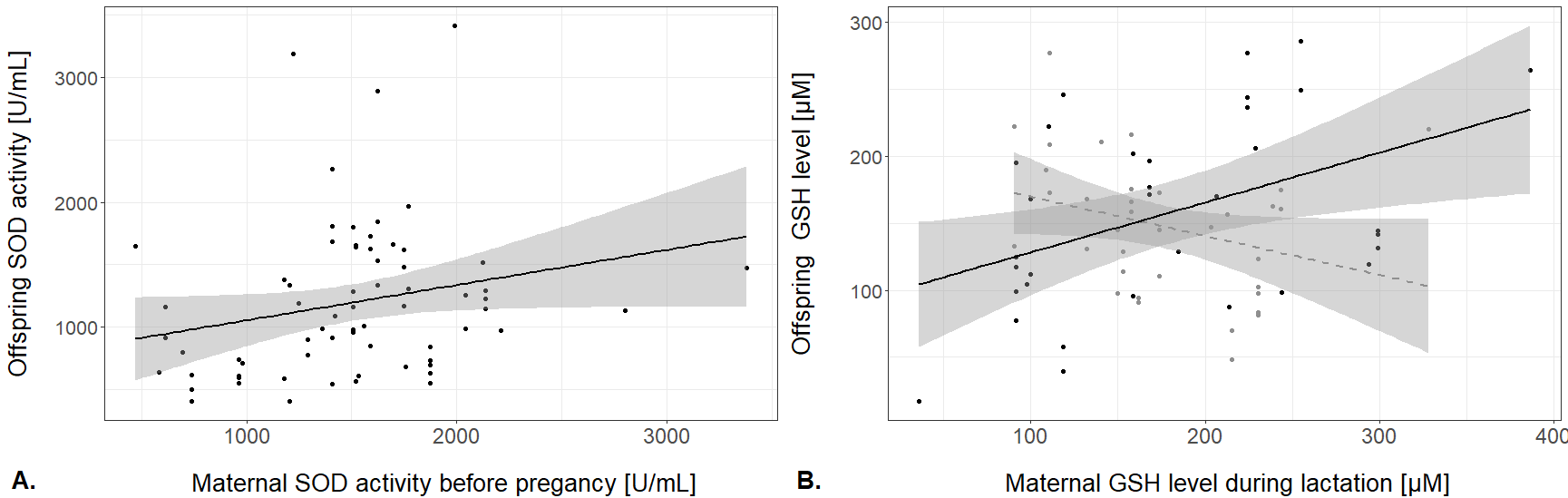


*Figure S2: Relationship between offspring oxidative stress marker levels and maternal oxidative stress marker levels according to treatment. The line represents the regression line +/- 95% confidence interval (shaded region) with points representing the raw data. Black dots and solid lines represent provisioned females, while grey dots and dashed lines represent non-provisioned females*

**Impact of experimental provisioning treatment on body mass**

To explore whether the experimental provisioning treatment impacted body mass, we ran a linear mixed model. Body mass measured before pregnancy was included as a response variable and treatment, time (before or after the experimental provisioning started) and their interaction were included as explanatory variables, with age at measurement included as a covariate. Maternal identity was included as a random effect.

All females significantly increased their body mass after the experimental provisioning started (Intercept: estimate +/- SE = 1439.46 +/- 71, Time: estimate +/- SE = -164.31 +/- 22.78, F-value _1,401.5_= 93, P-value<0.001) , regardless of their treatment (estimate +/- SE = -38.3 +/- 98.95, F-value _1,5.95_= 0.05, P-value= 0.83), and beyond the effect of age (estimate +/- SE = 75.05 +/- 19.16, F-value _1,11.56_= 15.34, P-value= 0.002). The interaction between treatment and time was not significant (estimate +/- SE = 29.71+/- 29.31, F-value _1,288.72_= 1.03, P-value= 0.31).


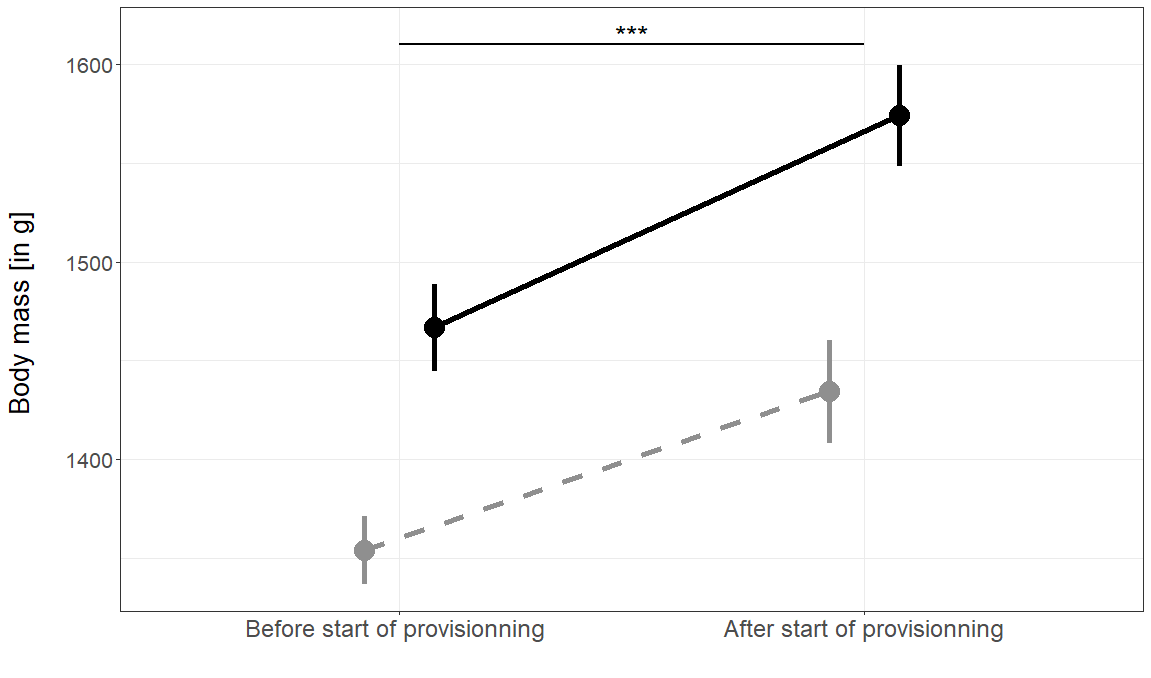


*Figure S3: Relationship between body mass and time (before or after start of provisioning). Symbols represent raw data means +/- SE Stars indicate statistical significance: ***: P-value<0.001. Black dots and solid lines represent provisioned females, while grey dots and dashed lines represent non-provisioned females.*

Table SII: Summary of models for each prediction tested

|  | **Prediction 1.1** | |  | | | **Prediction 1.2** | | | | |  | **Prediction 2.1** | | | | | | |  | **Prediction 2.2** | | | | | | | | | |
| --- | --- | --- | --- | --- | --- | --- | --- | --- | --- | --- | --- | --- | --- | --- | --- | --- | --- | --- | --- | --- | --- | --- | --- | --- | --- | --- | --- | --- | --- |
| Response variables | Oxidative stress markers | |  | | | Pre-natal investment  Offspring’ body mass  at emergence  Survival to emergence  Survival to 12 months | | | | |  | Changes in oxidative stress markers | | | | | | |  | Pre-natal investment  Offspring’ body mass  at emergence | | | | | | | | | |
| Explanatory variables |  |  | |  |  | |  |  |  | |  |  | | |  | | |  | | |  | | |  |  | |  | | |
|  | Provisioning treatment | |  | | | Offspring age at emergence | | | | |  | *Model 1:* | | | | | | |  | Offspring age at emergence | | | | | | | | | |
|  |  |  |  |  |  |  |  |  |  |  |  |  |  |  |  |  |  |  |  |  |  |  |  |  |  |  |  |  |  |
|  | Stage of reproduction | |  | | | Fetus number | | | | |  | Pre-natal investment | | | | | | |  | PC (before repro) | | | | | | | | | |
|  |  |  |  |  |  |  |  |  |  |  |  |  |  |  |  |  |  |  |  |  |  |  |  |  |  |  |  |  |  |
|  | Provisioning treatment x  Stage of reproduction | |  | | | Provisioning treatment | | | | |  | *Model 2:* | | | | | | |  | MDA (before repro) | | | | | | | | | |
|  |  |  |  |  |  |  |  |  |  |  |  |  |  |  |  |  |  |  |  |  |  |  |  |  |  |  |  |  |  |
|  |  | |  | | | Provisioning treatment x  Fetus number | | | | |  | Offspring body mass at  emergence corrected | | | | | | |  | | | SOD (before repro) | | | | | | | |
|  |  |  |  |  |  |  |  |  |  |  |  |  |  |  |  |  |  |  |  |  |  |  |  |  |  |  |  |  |  |
|  |  |  | |  |  | | | | | |  |  | | | | | | |  | | | GSH (before repro) | | | | | | | |
|  |  |  |  |  |  |  |  |  |  |  |  |  |  |  |  |  |  |  |  |  |  |  |  |  |  |  |  |  |  |

|  | **Prediction 2.3.1** | | |  | **Prediction 2.3.2** | | | | | |  | | **Prediction 2.3.3** | | |
| --- | --- | --- | --- | --- | --- | --- | --- | --- | --- | --- | --- | --- | --- | --- | --- |
| Response variables | Oxidative stress markers | | |  | Pre-natal investment  Offspring’ body mass  at emergence  Survival to emergence  Survival to 12 months | | | | | |  |  | Change in oxidative stress markers | | |
| Explanatory variables |  |  |  | | | |  |  |  |  | |  | | |  |
|  | Stage of reproduction | | |  | Offspring age at emergence | | | | | |  | | PC (before repro) | | |
|  |  |  |  |  |  |  |  |  |  |  |  |  |  |  |  |
|  | Breeding status | | |  | Number of foetuses | | | | | |  | | MDA (before repro) | | |
|  |  |  |  |  |  |  |  |  |  |  |  |  |  |  |  |
|  | Breeding status x  Stage of reproduction | | |  | PC (pregnancy) | | | | | |  | | SOD (before repro) | | |
|  |  |  |  |  |  |  |  |  |  |  |  |  |  |  |  |
|  |  | | |  | MDA (pregnancy) | | | | | |  | | GSH (before repro) | | |
|  |  |  |  |  |  |  |  |  |  |  |  |  |  |  |  |
|  |  |  |  |  |  | SOD (pregnancy) | | | |  | |  | | |  |
|  |  |  |  |  |  |  |  |  |  |  |  |  |  |  |  |
|  |  |  |  |  |  | GSH (pregnancy) | | | |  | |  | | |  |
|  |  |  |  | | |  | | | |  | |  | | |  |

Reference

Kelly C, Price TD (2005) Correcting for regression to the mean in behavior and ecology. Am Nat 166:700–707. https://doi.org/10.1086/497402

Nussey DH, Pemberton JM, Pilkington JG, Blount JD (2009) Life history correlates of oxidative damage in a free-living mammal population. Functional Ecology 23:809–817. https://doi.org/10.1111/j.1365-2435.2009.01555.x
